# Epinephrine during resuscitation of traumatic cardiac arrest and increased mortality: a post hoc analysis of prospective observational study

**DOI:** 10.1186/s13049-019-0657-8

**Published:** 2019-08-16

**Authors:** Ryo Yamamoto, Masaru Suzuki, Kei Hayashida, Jo Yoshizawa, Atsushi Sakurai, Nobuya Kitamura, Takashi Tagami, Taka-aki Nakada, Munekazu Takeda, Junichi Sasaki, on behalf of the SOS-KANTO 2012 Study Group

**Affiliations:** 1 Department of Emergency and Critical Care Medicine, Keio University School of Medicine, 35 Shinanomachi, Shinjuku, Tokyo, 160-8582 Japan; 2 Department of Emergency Medicine, Tokyo Dental College, Ichikawa General Hospital, 5-11-13 Sugano, Ichikawa, Chiba, 272-8513 Japan; 3 Division of Emergency and Critical Care Medicine, Department of Acute Medicine, Nihon University School of Medicine, 30-1 Oyagutikamichou, Itabashi, Tokyo, 173-8610 Japan; 4 Department of Emergency and Critical Care Medicine, Kimitsu Chuo Hospital, 1010 Sakurai, Kisarazushi, Chiba, 292-8535 Japan; 5 Department of Emergency and Critical Care Medicine, Nippon Medical School Tama Nagayama Hospital, 1-7-1 Nagayama, Tama-shi, Tokyo, 206-8512 Japan; 6 Department of Emergency and Critical Care Medicine, Chiba University Graduate School of Medicine, 1-8-1 Inohana, Chuo-ku, Chiba City, Chiba, 260-8677 Japan; 7 Department of Critical Care and Emergency Medicine, Tokyo Women’s Medical University, 8-1 Kawada-cho, Shinjuku, Tokyo, 162-8666 Japan

**Keywords:** Epinephrine, Out-of-hospital cardiac arrest, Trauma, Mortality

## Abstract

**Background:**

The beneficial effect of epinephrine during resuscitation from out-of-hospital cardiac arrest (OHCA) has been inconclusive, and potential harm has been suggested, particularly in trauma victims. Although no significant improvement in neurological outcomes has been found among resuscitated patients using epinephrine, including trauma patients, the use of epinephrine is recommended in the Advanced Trauma Life Support protocol. Given that the use of vasopressors was reported to be associated with increased mortality in patients with massive bleeding, the undesirable effects of epinephrine during the resuscitation of traumatic OHCA should be elucidated. We hypothesised that resuscitation with epinephrine would increase mortality in patients with OHCA following trauma.

**Methods:**

This study is a post-hoc analysis of a prospective, multicentre, observational study on patients with OHCA between January 2012 and March 2013. We included adult patients with traumatic OHCA who were aged ≥15 years and excluded those with missing survival data. Patient data were divided into epinephrine or no-epinephrine groups based on the use of epinephrine during resuscitation at the hospital. Propensity scores were developed to estimate the probability of being assigned to the epinephrine group using multivariate logistic regression analyses adjusted for known survival predictors. The primary outcome was survival 7 days after injury, which was compared among the two groups after propensity score matching.

**Results:**

Of the 1125 adults with traumatic OHCA during the study period, 1030 patients were included in this study. Among them, 822 (79.8%) were resuscitated using epinephrine, and 1.1% (9/822) in the epinephrine group and 5.3% (11/208) in the no-epinephrine group survived 7 days after injury. The use of epinephrine was significantly associated with decreased 7-day survival (odds ratio = 0.20; 95% CI = 0.08–0.48; *P* < 0.01), and this result was confirmed by propensity score-matching analysis, in which 178 matched pairs were examined (adjusted odds ratio = 0.11; 95% CI = 0.01–0.85; *P* = 0.02).

**Conclusions:**

The relationship between the use of epinephrine during resuscitation and decreased 7-day survival was found in patients with OHCA following trauma, and the propensity score-matched analyses validated the results. Resuscitation without epinephrine in traumatic OHCA should be further studied in a randomised controlled trial.

## Introduction

Epinephrine, an active sympathomimetic hormone stimulating the alpha- and beta-adrenergic systems [1], has been considered a major component of advanced life support for out-of-hospital cardiac arrest (OHCA) [2–5]. Since animal studies revealed that 1 mg of epinephrine improved survival of asphyxiated dogs in the 1960s [6], the American Heart Association Guidelines for Advanced Cardiac Life Support has recommended epinephrine administration for cardiac arrest (CA) with shockable or non-shockable rhythms [3] because aortic diastolic pressure is amplified with the alpha-adrenergic effect, thus leading to coronary perfusion augmentation [7].

While several investigators have attempted to elucidate the optimal dose and beneficial effects of epinephrine during cardiopulmonary resuscitation (CPR), the results have been inconclusive [5, 8–13]. A randomised controlled trial involving more than 8000 patients with OHCA identified no significant differences in favourable neurologic outcomes between patients treated with and without epinephrine, whereas epinephrine use resulted in a higher survival rate at 30 days [10]. Another randomised controlled trial investigating epinephrine for OHCA found no improvement in survival upon hospital discharge, whereas the likelihood of return of spontaneous circulation (ROSC) was improved in the epinephrine group [11].

Although epinephrine use for patients with traumatic OHCA has been reported in some regions [14, 15], and has been recommended in the Advanced Trauma Life Support protocol [16], potential harm was suggested in trauma victims [17–19]. A study evaluating resuscitation from major injuries revealed that vasopressor use was associated with increased mortality in patients with massive bleeding [17], and another study using an animal model with haemorrhagic shock found that epinephrine use was related to worse outcomes compared to fluid resuscitation [18]. It should also be emphasised that epinephrine administered to patients with traumatic OHCA was reported to have no significant association with survival upon discharge [15]. Given that there was no significant improvement in neurological outcomes among resuscitated trauma patients using epinephrine [20], potential harm from its use during CPR should be considered in trauma victims.

Accordingly, to elucidate the potential undesirable effects of epinephrine during resuscitation of patients with traumatic OHCA, the OHCA mortality rate after a major trauma was examined by using a post-hoc data analysis in a multicentre study on OHCA in Japan with a propensity score matching analysis to reduce the effects of confounding factors. We hypothesised that in-hospital resuscitation with epinephrine would increase mortality in patients with OHCA following trauma.

## Methods

### Study design and setting

A post-hoc data analysis was conducted on a prospective, multicentre, observational study (SOS-KANTO 2012) consisting of patients who suffered OHCA and were transported to 67 emergency hospitals by emergency medical service (EMS) personnel in the Kanto area, including Tokyo and its suburbs, between January 2012 and March 2013. SOS-KANTO 2012 was maintained with support from the Kanto chapter of the Japanese Association for Acute Medicine. Details on the design of the SOS-KANTO 2012 study are provided elsewhere [21, 22]. Data were prospectively collected by the treating physicians or volunteer registrars designated at each hospital.

In Japan, EMS personnel perform CPR according to the Japanese CPR guidelines, which were developed and revised based on the American Heart Association guidelines and International Liaison Committee on Resuscitation guidelines. Although most EMS crews have an emergency life-saving technician (ELST) who is certified to obtain intravenous access, only a specially trained ELST can administer epinephrine under instructions from a medical director in each region. No EMS personnel are authorised to perform Advanced Trauma Life Support interventions, such as intraosseous access or needle/tube thoracostomy.

### Selection of participants

Data from SOS-KANTO 2012 were retrospectively reviewed, and patients with OHCA following trauma were identified. Traumatic OHCA was diagnosed by treating physicians based on history of OHCA and/or clinical findings, and drowning and hanging were not considered traumatic OHCA. The inclusion criteria were as follows: patients aged ≥15 years with available data on epinephrine administration during resuscitation after hospital arrival. Patients with missing or unknown survival data 7 days after injury were excluded.

### Interventions and other data definitions

The intervention in this study was defined as epinephrine administration during resuscitation at hospitals, which was recorded as epinephrine use after hospital arrival in the database. Epinephrine use for patients with spontaneous circulation was not considered an intervention examined in this study. Since there was no guideline clearly indicating epinephrine use for patients with traumatic OHCA, administration of epinephrine during in-hospital resuscitation was decided clinically by the treating physicians. Epinephrine use prior to hospital arrival was not considered an intervention because pre-hospital administration of epinephrine could vary depending on the EMS provider status and/or pre-hospital health system across regions in the study population rather than based on patient status or decisions by treating health care providers.

Other available data included age, sex, injury mechanism, witness status, presence of bystander CPR, presence of signs of life at the scene, initial cardiac rhythm, time of emergency call, time of ambulance arrival, time of CPR initiation by EMS personnel, epinephrine administration prior to hospital arrival, ROSC prior to hospital, time of hospital arrival, presence of signs of life on arrival, cardiac rhythm on arrival, ROSC in the hospital and survival status at 7 days after injury. Pre-hospital information were prospectively collected by EMS providers in the standardised Utstein style [21, 22]. In-hospital information was collected by treating physicians at each institution, and survival information were collected by phone survey if patients were discharged from the hospital or transferred to another hospital. CPR duration until arrival was defined as the interval between CPR initiation by the EMS personnel and hospital arrival. Signs of life were defined as the presence of any of the following: spontaneous respirations, palpable pulse, measurable blood pressure, spontaneous movement or pupillary reactivity. Other variables related to the resuscitation of patients with traumatic OHCA, such as time of identification of haemorrhagic injury, time and type of definitive or damage control surgery, amount of fluid resuscitation, and amount of transfusion, were not available in the database [21, 22].

### Measurements

The primary outcome was the survival rate 7 days after injury, which was selected as a surrogate marker for vital clinical outcomes, such as 30-day mortality, to maximise capturing the effects of epinephrine use. The secondary outcomes were the ROSC rate at the hospital, recorded as arrival with spontaneous circulation, or ROSC after hospital arrival, including non-survivors with temporally sustained spontaneous circulation.

### Statistical analysis

Patients were divided into epinephrine and no-epinephrine groups. The epinephrine group consisted of patients administered with epinephrine during in-hospital resuscitation, whereas the no-epinephrine group consisted of those who were treated without epinephrine administration.

As several cofounders can affect survival after injury, propensity score matching was performed to compare the primary outcome between groups and to assess secondary outcomes [23]. A multivariate logistic regression was used to determine propensity scores to predict the probability of being assigned to the epinephrine group compared with the no-epinephrine group. Relevant covariates were carefully selected from known survival predictors in traumatic OHCA, such as age, witness status, bystander CPR, signs of life at the scene, and CPR duration until hospital arrival, and entered into the propensity model to ensure high-fidelity propensity scores [1–5, 24–27]. Possible cofounding factors that could be related to epinephrine use during in-hospital resuscitation, such as signs of life upon arrival at the hospital and ROSC achieved prior to hospital arrival, were also entered into the model. Although epinephrine use prior to hospital arrival and time from recognition of CA to the initial dose of pre-hospital epinephrine might not affect the decision to administer epinephrine in-hospital, these variables were entered into the propensity score calculation to balance the distribution of these variables between the two groups. Patients with missing covariates were excluded from the propensity score calculation. The precision of discrimination and propensity score calibration was analysed using the c-statistic and Hosmer–Lemeshow goodness-of-fit test. Propensity score matching extracted one-to-one matched pairs of patients with a nearest-neighbour matching algorithm, in which the calliper width was adjusted to preserve the effect size of matched pairs [23, 28, 29].

An intergroup comparison between primary and secondary outcomes after propensity score matching was performed using linear regression analysis. Then, sensitivity analyses were performed to validate the primary results. To confirm that the results were not dependent on the matching method, inverse probability weighting and logistic regression analyses were performed (using the propensity score as a covariate in the logistic regression analysis) for 7-day survival after the injury. Furthermore, multivariate logistic regression was performed using all patient data before propensity score calculations to assess the robustness of the study results.

Several subgroup analyses were also performed to evaluate the heterogeneity of patients with OHCA after injury. Given that epinephrine administration prior to hospital arrival could modify the effects of in-hospital epinephrine, one of the selected subgroups included patients who were not administered epinephrine prior to hospital arrival. Another subgroup consisted of patients who arrived at the hospital without any signs of life because the presence of signs of life upon arrival is a significant survival predictor in trauma victims. Primary and secondary outcomes were compared between the epinephrine and no-epinephrine groups in the selected patients using univariate analyses.

Descriptive statistics were presented as the means ± standard deviation, median (interquartile range), or number (%). The results were compared using unpaired t-tests, Mann–Whitney U tests, Chi-square tests or Fisher’s exact tests, as appropriate. To test all hypotheses, a two-sided α threshold of 0.05 was considered statistically significant. All statistical analyses were conducted using IBM SPSS Statistics, version 24.0 (IBM, Armonk, NY, USA) and Microsoft Excel (Microsoft, Redmond, WA, USA).

## Results

**Fig. 1:**
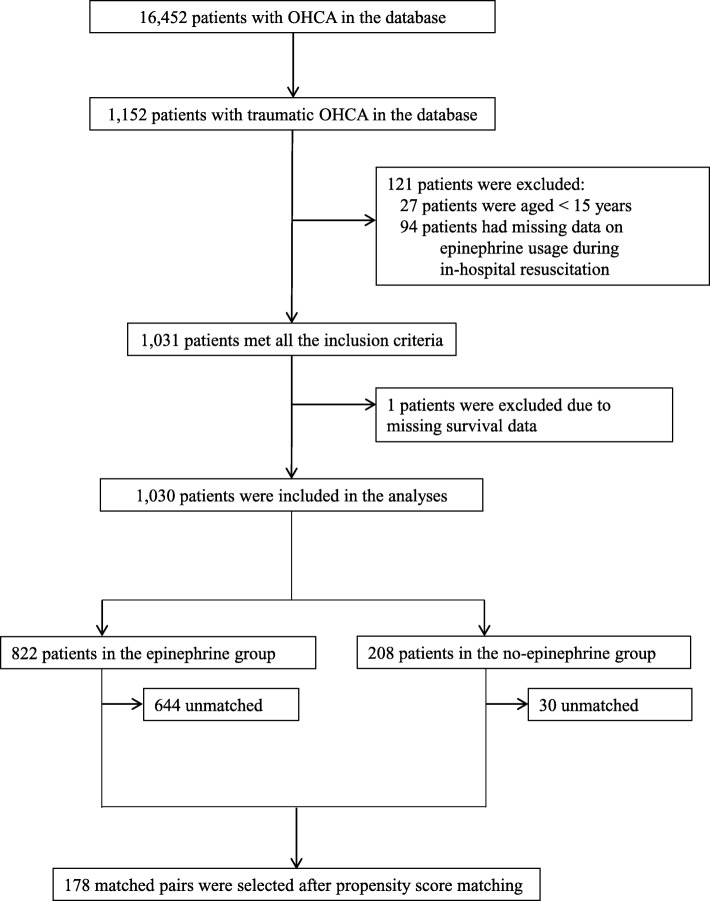
Flow diagram of the study. A total of 1152 patients with traumatic OHCA were identified; among them, 1030 were included in the analyses, and 356 (178 pairs) were identified in the propensity score matching. Abbreviations: OHCA = out-of-hospital cardiac arrest

After the screening process, 1152 patients with traumatic OHCA who presented to collaborating hospitals during the study period were identified. Among the 1125 people aged ≥15 years, 94 had no information on epinephrine administration during in-hospital resuscitation. Although 1031 patients satisfied all the inclusion criteria, one was excluded due to missing survival data. The patient flow diagram is shown in Fig. 1.

Finally, 1030 patients were eligible for this study: 822 (79.8%) patients were resuscitated with epinephrine administration after hospital arrival, and 208 (20.2%) were not treated with epinephrine at hospitals. The patient characteristics are summarised in Table 1. More patients in the epinephrine group received pre-hospital epinephrine than those in the no-epinephrine group (94 [12.2%] vs. 11 [5.5%], respectively). The median time from the recognition of CA to the initial dose of pre- and in-hospital epinephrine was 14 [2–26] and 28 [14–32] mins, respectively, in the epinephrine group, whereas these data were missing in the no-epinephrine group. The number of patients in the epinephrine group who had signs of life at the scene was higher than the no-epinephrine group (84 [10.2%] vs. 8 [3.9%], respectively), as were those who became pulseless during transportation (97 [12.0%] vs. 12 [5.8%]). The number of patients who regained spontaneous circulation before hospital arrival (23 [2.9%] vs. 15 [7.2%], respectively) and had signs of life upon hospital arrival (25 [3.1%] vs. 21 [10.1%]) was lower in the epinephrine group. Mechanism of injury (penetrate vs blunt) was comparable between the groups (penetrating injuries = 50 [8.4%] vs. 9 [7.8%]). The interval between the emergency call and ambulance arrival at the scene was also statistically comparable between the two groups, whereas CPR duration was shorter in the epinephrine group.

**Table 1:** Standardised differences of patients with traumatic OHCA

	Before Matching					After Matching				
	Epinephrine		no-Epinephrine		Standardised Difference	Epinephrine		no-Epinephrine		Standardised Difference
Case	822		208			178		178		
Age, years, median (IQR)	54	(33)	54	(30)	4.4%	55	(30)	54	(31)	2.5%
missing data	59	(7.2%)	18	(8.7%)						
Sex, male, n (%)	581	(70.7%)	133	(63.9%)	14.4%	124	(70.0%)	109	(61.2%)	17.8%
missing data	0	(0%)	0	(0%)						
Witness, n (%)	482	(58.8%)	110	(52.9%)	11.9%	102	(57.3%)	90	(50.6%)	13.6%
missing data	2	(0.2%)	0	(0%)						
Bystander CPR, n (%)	133	(16.3%)	25	(12.1%)	12.1%	26	(14.6%)	18	(10.1%)	13.7%
missing data	5	(0.6%)	1	(0.5%)						
Signs of life at scene, n (%)	84	(10.2%)	8	(3.9%)	25.0%	12	(6.7%)	6	(3.4%)	15.4%
missing data	0	(0%)	1	(0.5%)						
Asystole at scene, n (%)	519	(64.4%)	161	(77.4%)	28.9%	132	(74.2%)	143	(80.3%)	14.8%
missing data	16	(1.9%)	0	(0%)						
Collapsed to CA during transportation, n (%)	97	(12.0%)	12	(5.8%)	21.8%	15	(8.4%)	8	(4.5%)	16.1%
missing data	11	(1.3%)	1	(0.5%)						
Epinephrine prior to hospital arrival, n (%)	94	(12.2%)	11	(5.5%)	23.8%	15	(8.4%)	10	(5.6%)	11.0%
missing data	54	(6.6%)	9	(4.3%)						
ROSC prior to hospital arrival, n (%)	23	(2.9%)	15	(7.2%)	20.0%	7	(3.9%)	8	(4.5%)	2.8%
missing data	19	(2.3%)	0	(0%)						
Signs of life on hospital arrival, n (%)	25	(3.1%)	21	(10.1%)	28.5%	12	(6.7%)	13	(7.3%)	2.2%
missing data	21	(2.6%)	1	(0.5%)						
Asystole on hospital arrival, n (%)	666	(81.8%)	181	(87.4%)	15.6%	157	(88.2%)	160	(89.9%)	5.4%
missing data	8	(1.0%)	1	(0.5%)						
Mechanism of Injury, penetrate, n (%)	50	(8.4%)	9	(7.8%)	2.0%	9	(5.1%)	9	(5.1%)	0.0%
missing data	225	(27.4%)	93	(44.7%)						
Emergency call to ambulance arrival, mins, median (IQR)	7	(4)	7	(4)	0.8%	7	(5)	7	(4)	5.7%
missing data	59	(7.2%)	18	(8.7%)						
CPR duration before hospital arrival, mins, median (IQR)	21	(11)	22	(10)	20.7%	21	(11)	22	(10)	13.9%
missing data	59	(7.2%)	18	(8.7%)						

*OHCA* Out-of-Hospital Cardiac Arrest, *CPR* Cardiopulmonary Resuscitation, *CA* Cardiac Arrest, *ROSC* Return of Spontaneous Circulation

Considering these non-negligibly biased distributions in the known survival predictors of patients with traumatic OHCA, propensity score matching was performed. The final propensity model predicting allocation in the epinephrine group included covariates such as age, presence of signs of life at the scene and/or upon hospital arrival, no electrical activity on cardiac rhythm (asystole) at the scene and/or upon hospital arrival, witness status, presence of bystander CPR, collapsed to CA during transportation, ROSC achieved prior to arrival, time from emergency call to ambulance arrival at the scene, CPR duration until hospital arrival, and administration of epinephrine before hospital arrival. Since time from the recognition of CA to the initial dose of pre-hospital epinephrine was missing among patients in the no-epinephrine group, this variable was not included in the final propensity model. The final model was shown to have sufficient discrimination and calibration for the probability of being assigned to the epinephrine group (c-statistic = 0.681 and Hosmer–Lemeshow goodness-of-fit *p* = 0.909).

Among the 822 patients in the epinephrine group, 178 matched with those in the no-epinephrine group. The patient characteristics after matching are summarised with standardised differences in covariates before and after matching in Table 1. Propensity score matching analysis revealed that survival 7 days after injury was significantly lower in patients resuscitated with epinephrine than those without epinephrine (1 [0.6%] vs. 9 [5.1%]; odds ratio [OR] = 0.11; 95% confidence interval [CI] = 0.01–0.85; *p* = 0.02; Table 2), but the proportion of patients who achieved ROSC in the hospital was higher in the epinephrine group than the no-epinephrine group (32 [18.0%] vs. 16 [9.0%]; OR = 2.21; 95% CI = 1.16–4.19; *p* = 0.01; Table 2).

**Table 2:** Impact of in-hospital epinephrine on 7-day survival and secondary outcomes

	Epinephrine	no-Epinephrine	*P* value	OR	95% CI
Unadjusted analyses					
Survival at 7 days, *n (%; 95%CI)*	9 (1.1%; 0.4–1.8%)	11 (5.3%; 2.2–8.3%)	< 0.01	0.20	0.08–0.48
ROSC, *n (%; 95%CI)*	144 (17.5%; 14.9–20.1%)	24 (11.6%; 7.2–15.9%)	0.04	1.62	1.02–2.57
Propensity score matching					
Survival at 7 days, *n (%; 95%CI)*	1 (0.6%; 0.0–1.7%)	9 (5.1%; 1.8–8.3%)	0.02	0.11	0.01–0.85
ROSC, *n (%; 95%CI)*	32 (18.0%; 12.3–23.6%)	16 (9.0%; 4.8–13.2%)	0.01	2.21	1.16–4.19

*ROSC* Return of Spontaneous Circulation

**Fig. 2:**
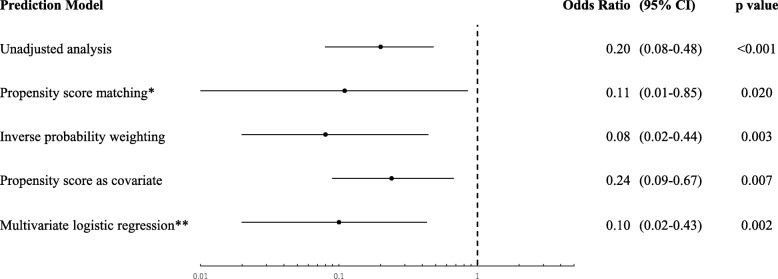
Effects of in-hospital epinephrine on 7-day survival. In-hospital epinephrine use was independently associated with lower 7-day survival (odds ratio = 0.11; 95% CI = 0.01–0.85; *p* = 0.02) in the propensity score-matched analysis, which was conducted as a primary analysis. To confirm that the results were not dependent on the matching method, inverse probability weighting and logistic regression analyses were performed (using the propensity score as the covariate in the logistic regression analysis). * Indicates primary analysis. ** Indicates multivariate logistic regression conducted before performing the propensity score matching. Abbreviations: CI = Confidence interval

Several sensitivity analyses were performed on the full population (1030 patients) including those excluded at the propensity score matching analyses. Inverse probability weighting analysis confirmed that epinephrine use during in-hospital resuscitation was significantly associated with a lower rate of survival 7 days after injury (OR = 0.08; 95% CI = 0.02–0.44; *p* = 0.003; Fig. 2), and logistic regression with the propensity score as a covariate validated that the association between epinephrine administration and mortality was not dependent on the matching method used (OR = 0.24; 95% CI = 0.09–0.67; *p* = 0.007). Furthermore, the multivariate logistic regression with all patient data before propensity score calculation revealed similar results (OR = 0.10; 95% CI = 0.02–0.43; *p* = 0.002).

Subgroup analyses were performed to evaluate the heterogeneity of patients with traumatic OHCA. Among the patients who were not administered epinephrine prior to hospital arrival, lower survival 7 days after injury was observed in the epinephrine group, but this result was not statistically significant (1 [0.6%] vs. 6 [3.6%]; OR = 0.17; 95% CI = 0.02–1.40; *p* = 0.12). Another subgroup analysis showed that none of the patients with no signs of life upon hospital arrival were alive 7 days after injury in the epinephrine group compared with two patients in the no-epinephrine group (0 [0.0%] vs. 2 [1.2%]; *p* = 0.25; Table 3). The proportion of patients who achieved ROSC in the hospital was significantly higher in the epinephrine group than the no-epinephrine group in these subgroup analyses (Table 3).

**Table 3:** In-hospital epinephrine in Subgroup Analyses

	Epinephrine	no-Epinephrine	*P* value	OR	95% CI
No signs of life upon arrival	166	165			
Survival at 7 days, *n (%; 95%CI)*	0 (0.0%; 0.0–0.0%)	2 (1.2%; 0.0–2.9%)	0.25	N/A	N/A
ROSC, *n (%; 95%CI)*	26 (15.7%; 10.1–21.2%)	5 (3.0%; 0.4–5.6%)	< 0.01	5.91	2.21–15.79
No epinephrine prior to arrival	163	168			
Survival at 7 days, *n (%; 95%CI)*	1 (0.6%; 0.0–1.8%)	6 (3.6%; 0.8–6.4%)	0.12	0.17	0.02–1.40
ROSC, *n (%; 95%CI)*	27 (16.6%; 10.9–22.3%)	9 (5.4%; 2.0–8.8%)	< 0.01	3.49	1.58–7.67

*ROSC* Return of Spontaneous Circulation

## Discussion

Propensity score matching was used to determine that epinephrine use during in-hospital resuscitation was independently associated with decreased survival 7 days after injury in patients with OHCA following trauma. Notably, the relationship was consistent based on several sensitivity analyses, indicating that the results were not dependent on the matching method, propensity scores, or statistical approach.

Although the reason behind the relationship between epinephrine use and increased mortality remains inconclusive, several pathophysiological mechanisms could be considered based on the undesirable effects of epinephrine, such as increased myocardial oxygen demand, arrhythmogenesis and cerebral arteriole vasoconstriction [1, 10, 30–32]. A large observational study revealed that epinephrine was associated with worse functional recovery after resuscitation from OHCA, and harmful epinephrine-induced reductions in microvascular blood flow were suggested in patients with ventricular fibrillation [30]. Another prospective cohort study found that a bolus of epinephrine during CPR produced no significant increase in cerebral oxygenation measured using cerebral oximetry [31]. An animal study using a CA model also found that epinephrine decreased the cerebral cortical microcirculatory blood flow [31]. Furthermore, 28 min was the median time from the recognition of CA to the initial dose of in-hospital epinephrine in this study, suggesting that epinephrine might have been given after the period when vasoconstriction would be most effective to maintain coronary perfusion.

The potential harm of epinephrine use found in this study was similarly reported in studies on critically injured patients [15, 17–19, 33]. An observational study revealed that vasopressor administration, including epinephrine, within 24 h of admission was independently associated with mortality regardless of fluid status [17]. Another retrospective cohort study investigating patients with haemorrhagic shock found that vasopressor use was related to reduce in-hospital survival after adjusting for trauma severity and volume of fluid resuscitation [19]. It should also be noted that a systematic review on hypotensive resuscitation in trauma victims suggested potential harm from interim increased blood pressure, which was attributed to increased bleeding rate [33].

Although the lower survival rate was suggested to be an undesirable harm of epinephrine use in this study, a higher incidence of ROSC was also observed among patients resuscitated with epinephrine. Since early death after severe trauma most commonly results from massive bleeding [14, 34, 35], the effects of epinephrine on regaining spontaneous circulation would not persist without haemostasis and might be outweighed by simultaneous undesirable effects, such as bleeding enhancement or cerebral arteriole vasoconstriction. ROSC achieved by vasopressor exposure should not be considered a goal of resuscitation, and physicians should avoid having false reassurance, particularly in patients with traumatic OHCA.

The results in this study must be interpreted within the context of the study design. Epinephrine use was only assessed for in-hospital resuscitation, rather than for pre- and in-hospital administration, because the former can vary depending on the EMS provider status and/or pre-hospital health system across regions or countries [36–39]; results for pre-hospital resuscitation may limit the generalisability of this study. Although the results might have been modified by epinephrine use during pre-hospital resuscitation, more than 90% of patients in this study were not treated with epinephrine prior to hospital arrival, and the propensity score matching adjusted the distribution of these covariates. The subgroup analyses of patients resuscitated without epinephrine administration before hospital arrival also showed that only one patient survived among the patients treated with epinephrine during in-hospital resuscitation.

Another limitation of this study is the fact that only a short-term outcome was considered the primary outcome, rather than long-term outcomes, such as 30-day survival or neurological outcomes 90 days after the injury. Our results might have overestimated the undesirable effects of epinephrine if the survival rate decreases at 30 days in both patients resuscitated with and without epinephrine use [40]. However, given that the survival after OHCA following trauma has been reported to be extremely rare [24, 25], we believe that decreased survival 7 days after injury would still show the relationship between epinephrine and unfavourable clinical outcomes. It should also be recognised that the survival rate at 7 days in patients treated with epinephrine in this study (0.6%) was lower than that at 30 days in patients who were not administered epinephrine as reported in other studies (3–5%) [14, 24, 25, 38].

Furthermore, imbalances between epinephrine and no-epinephrine groups remained in some variables after propensity score matching [23]. Although biased distributions might have affected the results, the underlying characteristics potentially favoured the epinephrine group, such as more witnessed injuries, shorter CPR duration before hospital arrival, and more frequent presence of signs of life at the scene, whereas 7-day survival was lower in this group. Notably, inverse probability weighting analysis and logistic regression with propensity score as a covariate validated the robustness of the results without using the matching procedure.

Finally, because this study was a retrospective study, the results were inconclusive. As some important variables related to resuscitation of patients with traumatic OHCA, such as time and type of diagnosis of haemorrhagic injury, time and type of surgical intervention, time to damage control surgery, amount of fluid resuscitation, and amount of transfusion, were not available in the database, the possible differences in the quality of the entire process of resuscitation between the groups might exist. Residual cofounding and unmeasured survival predictors, such as presence of devastating traumatic brain injury, were also impediments in confirming the association between epinephrine use and increased mortality. Therefore, the results do not provide definite conclusions and would only provide rationale for further research. Additional clinical investigations, including a prospective observational study, must be conducted to validate our results.

## Conclusions

In conclusion, epinephrine administered during in-hospital resuscitation was associated with lower 7-day survival rate in patients with OHCA following trauma. While we examined the outcomes of selected patients with limited data that were lacking critical information related to resuscitation of injured patients, we recommend that epinephrine should be deliberately used during the resuscitation of patients with traumatic OHCA. Further studies should be undertaken to confirm the unfavourable effects of epinephrine among patients with traumatic OHCA.

## Data Availability

The data in this study are available from the Kanto Local Association, Japanese Association for Acute Medicine, but restrictions apply to its availability, as the data were used under licence for the current study and are not publicly available. However, data are available from the authors upon reasonable request and with permission from the Kanto Local Association, Japanese Association for Acute Medicine.
